# Supplementary material for: A non-dispersion strategy for large-scale production of ultra-high concentration graphene slurries in water
Source: Nat Commun. 2018 Jan 8;9:76. doi: 10.1038/s41467-017-02580-3 (PMC5758749; doi:10.1038/s41467-017-02580-3)
Supplement: Supplementary file 1 — Supplementary Information [file 41467_2017_2580_MOESM1_ESM.pdf]

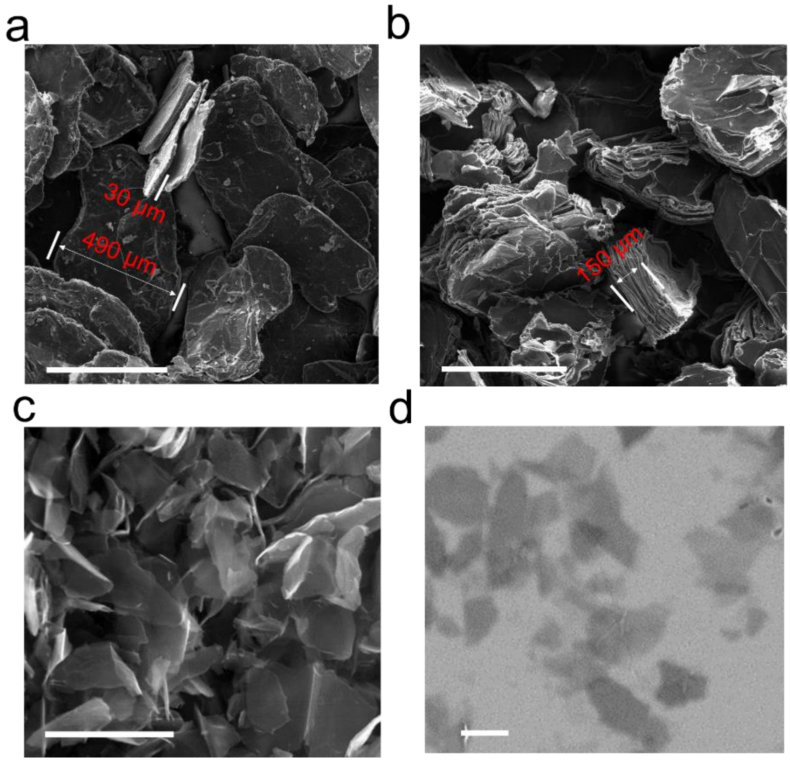

Supplementary Figure 1. SEM image of graphite (a), partially oxidized graphite (b), dry graphene powder (c), and graphene flakes (d). Scale bar: a-b. 500 µm; c. 2 µm; d. 1 µm.

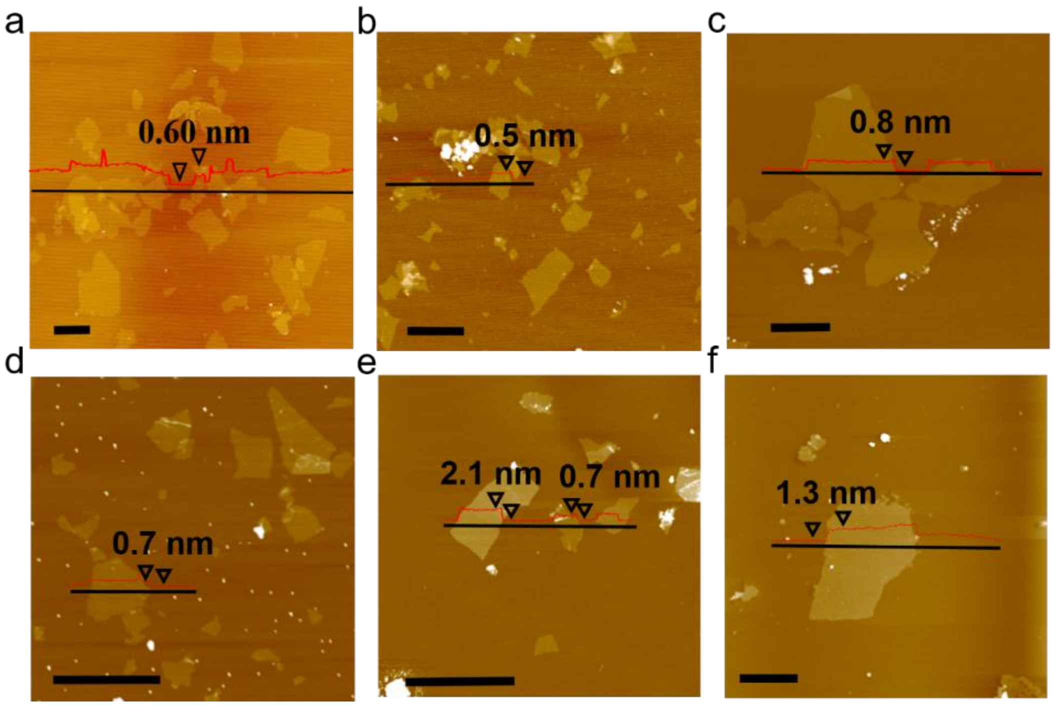

Supplementary Figure 2. Representative AFM images of graphene flakes by high-speed shear. Scale bar, 2 μm.

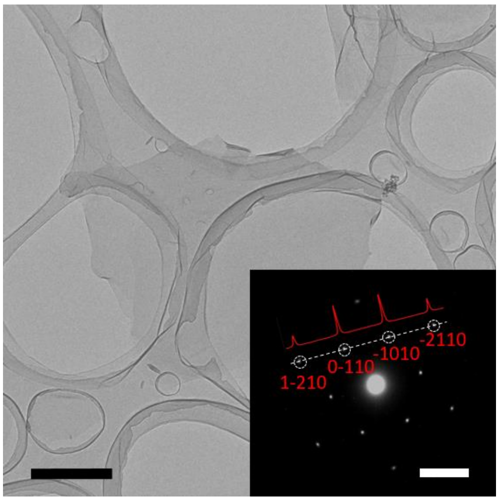

Supplementary Figure 3. TEM image and the corresponding SAED pattern of single-layer graphene sheet. Scale bar: 1 μm; Inset. 5 nm^{-1}.

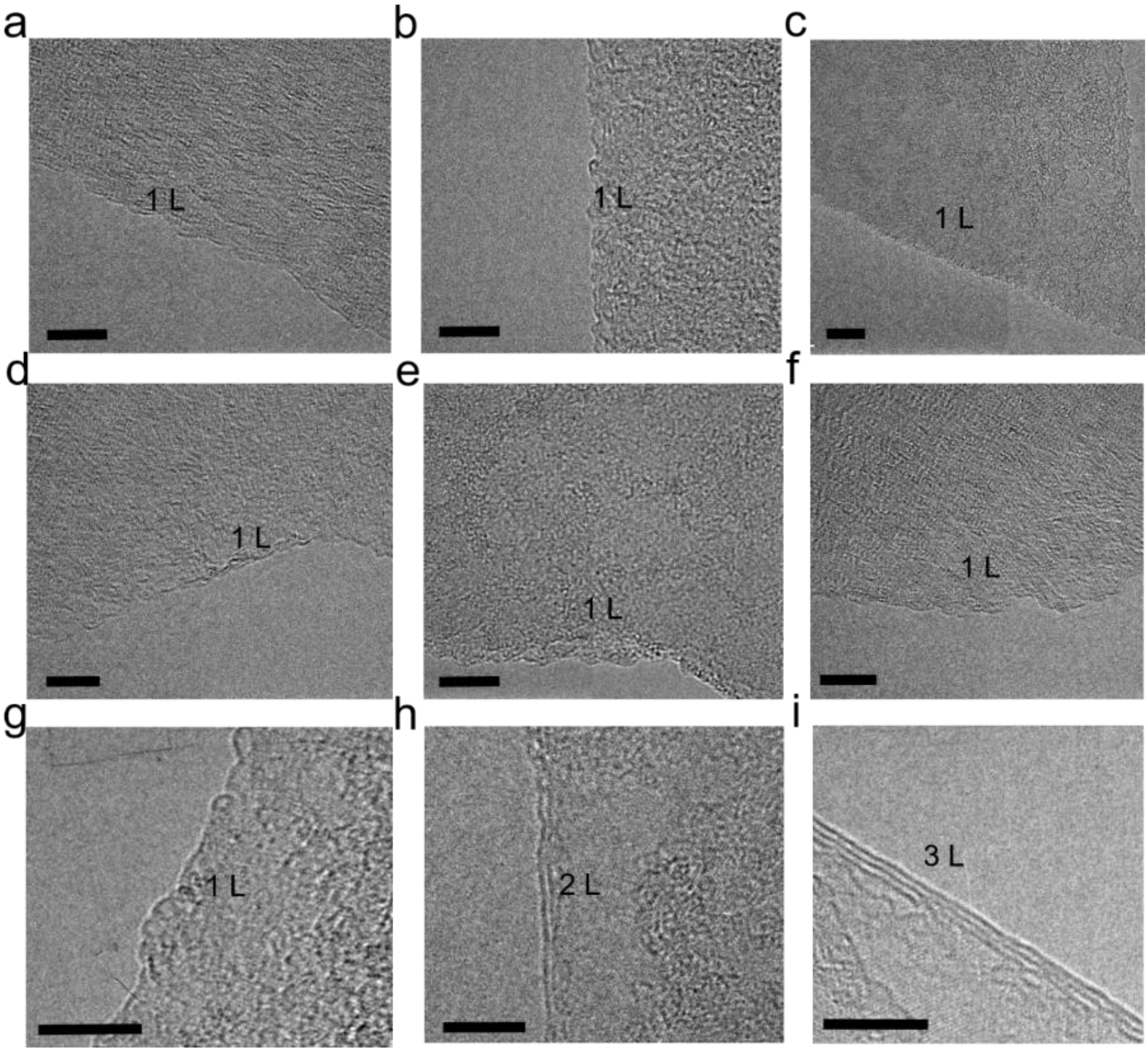

Supplementary Figure 4. (a-i) HRTEM images of graphene sheets. Scale bar, 5 nm.

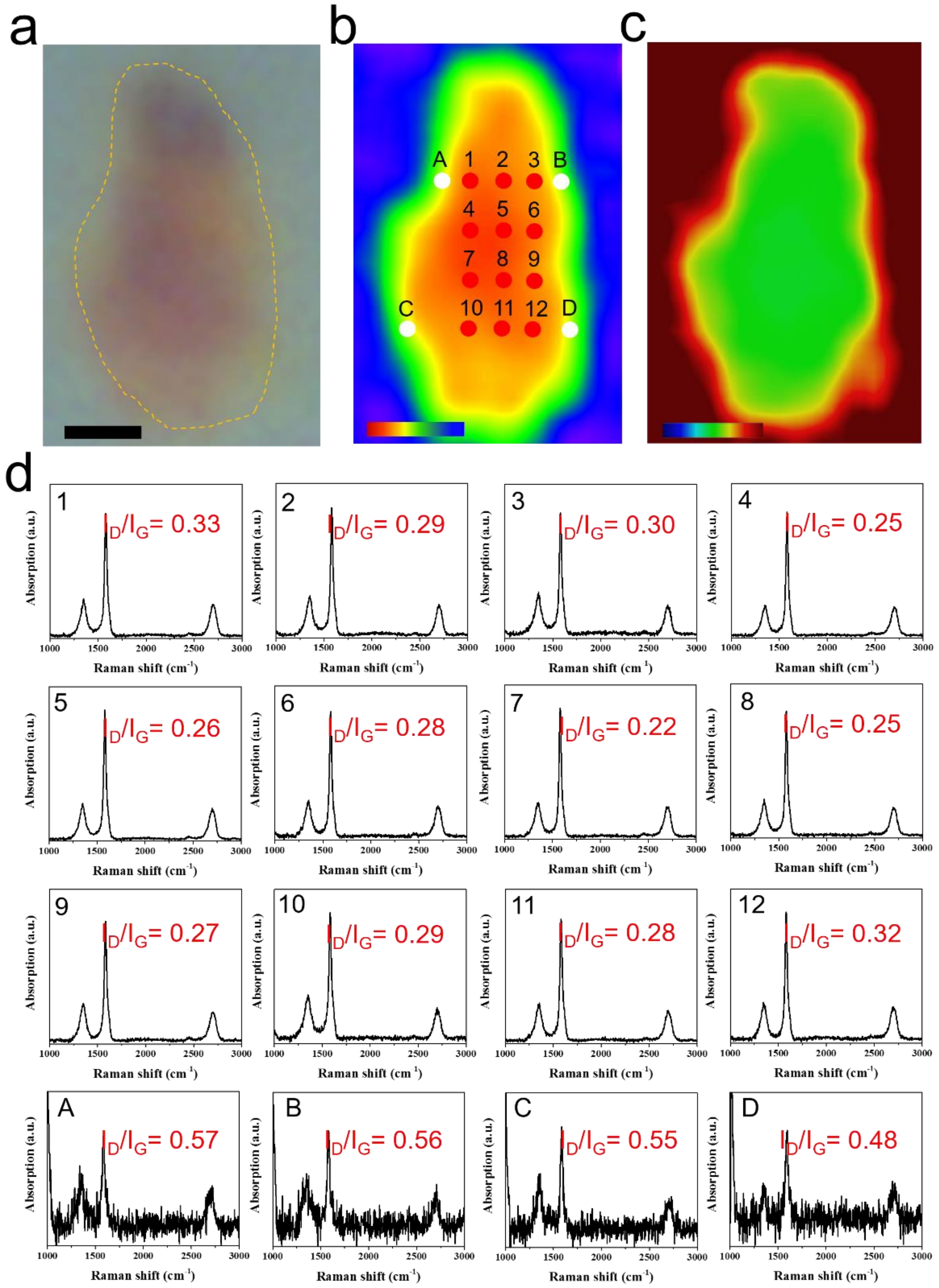

Supplementary Figure 5. Raman characterization of an isolated graphene flake. Optical image (a) and corresponding I_D/I_G mapping (b) and I_{2D}/I_G mapping (c) confirm the homogeneous structure of the graphene flake. Scale bar: a. 2 μm; b. 0-1 (I_D/I_G ratio); c. 0-1 (I_{2D}/I_G ratio); (d) Raman spectra of the corresponding 16 measured points in (b). The I_D/I_G ratio at the edge is higher, compared with those inner points.

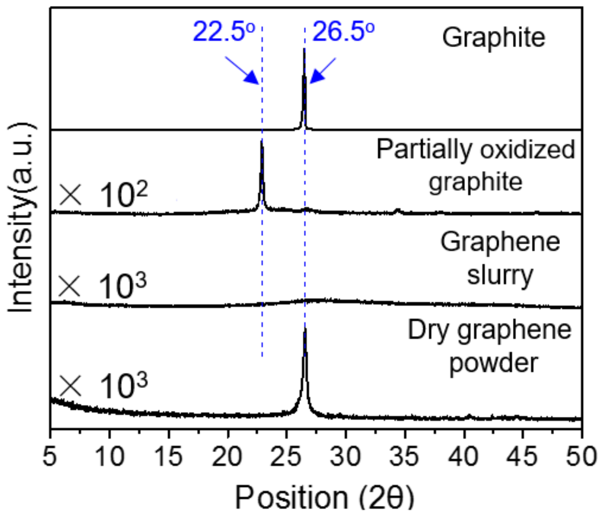

Supplementary Figure 6. XRD spectra of graphite, partially oxidized graphite (stage 1-graphite intercalated compound), graphene slurry and dry graphene powder.

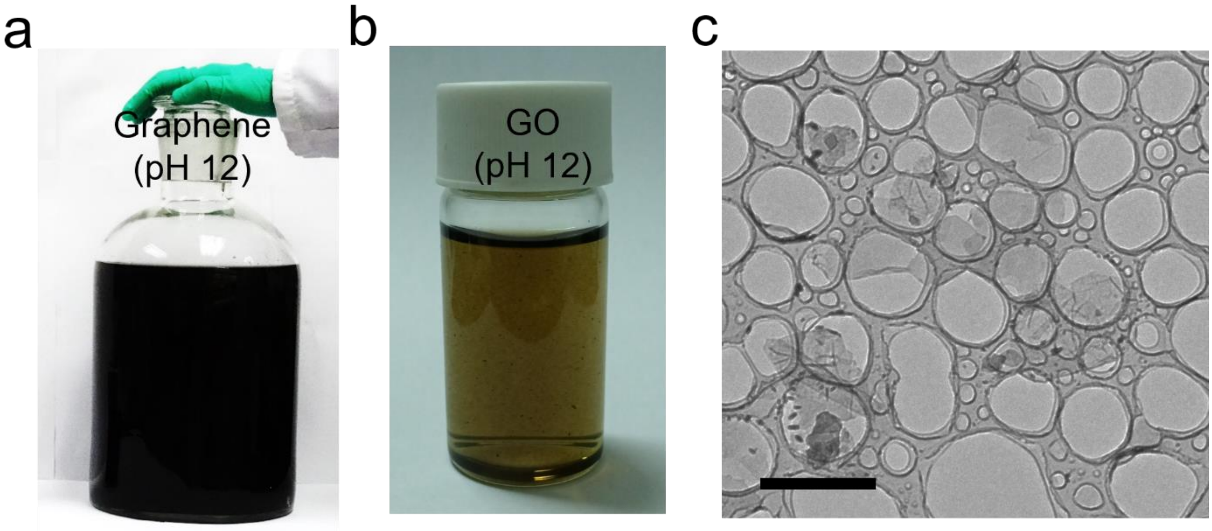

Supplementary Figure 7. (a,b) Digital photos of graphene and GO dispersion at pH = 12 after storing for 1 week, both at 0.1 mg mL^{-1}. (c) TEM image of graphene dispersion at pH = 12 after 1 week. Scale bar: c. 5 µm.

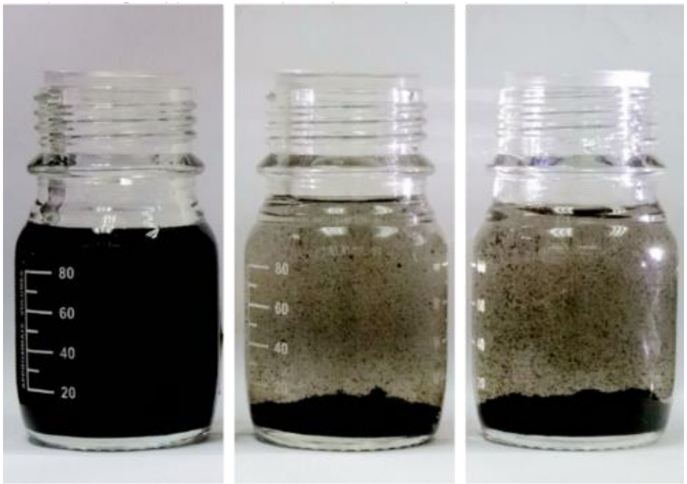

Supplementary Figure 8. Flocculation by adding NaOH (middle) or NaCl (right) into a graphene dispersion (pH = 12, left).

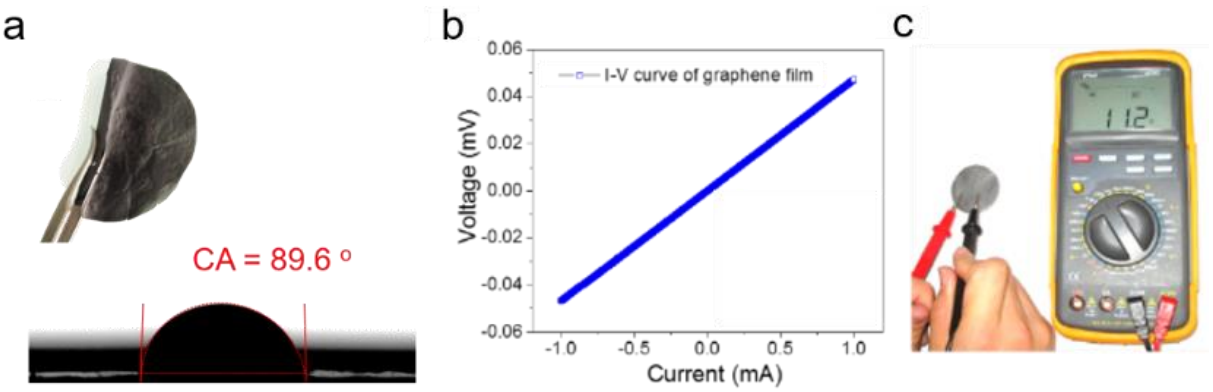

Supplementary Figure 9. (a) Water contact angle (CA) of graphene film. (b) I-V curve of graphene film by four-point probe meter. (c) Resistance measurement of graphene film by a multimeter, showing a low resistance of 11.2 Ω. The graphene film is prepared as follows: the graphene slurry was repeatedly centrifuged/washed to remove the residual ions. Neutral graphene flakes were then re-dispersed in NMP by ultrasonic treatment for 10 min. The graphene-NMP dispersion was filtered with a PTFE membrane (0.22 μm). Graphene film was peeled off from the PTFE membrane after drying.

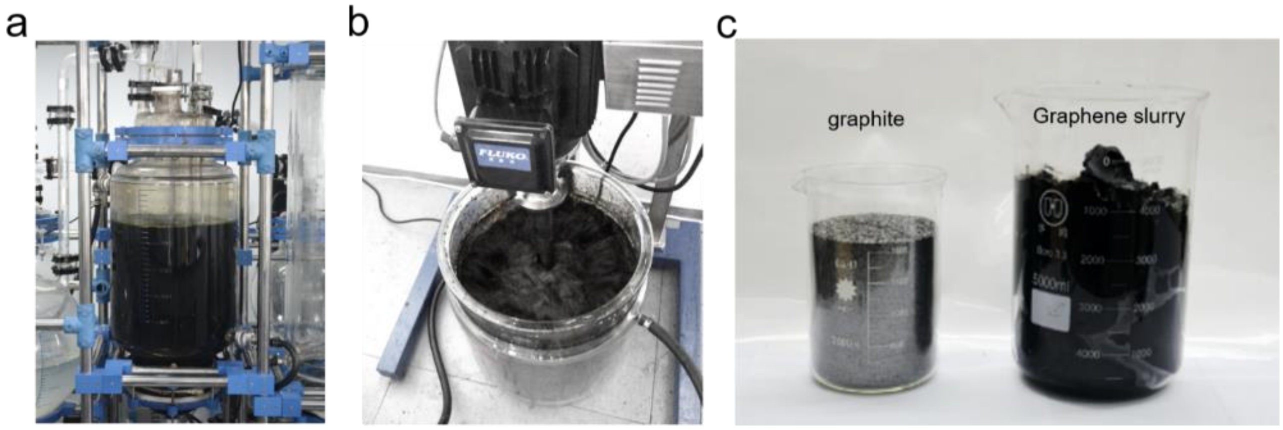

Supplementary Figure 10. (a) Intercalation process of 1 kg graphite (by four batches). (b) Exfoliation process of 1 kg graphite in an alkaline aqueous solution (20 L, pH = 14). (c) 1 kg raw graphite (left) and the corresponding highly concentrated slurry (right). Graphene slurry (~ 50 mg mL^{-1}) was prepared after shearing for 4 h, with yields of 68.2 wt% (mass ratio relative to raw graphite) and ~ 82.0 % (statistical value from the AFM images).

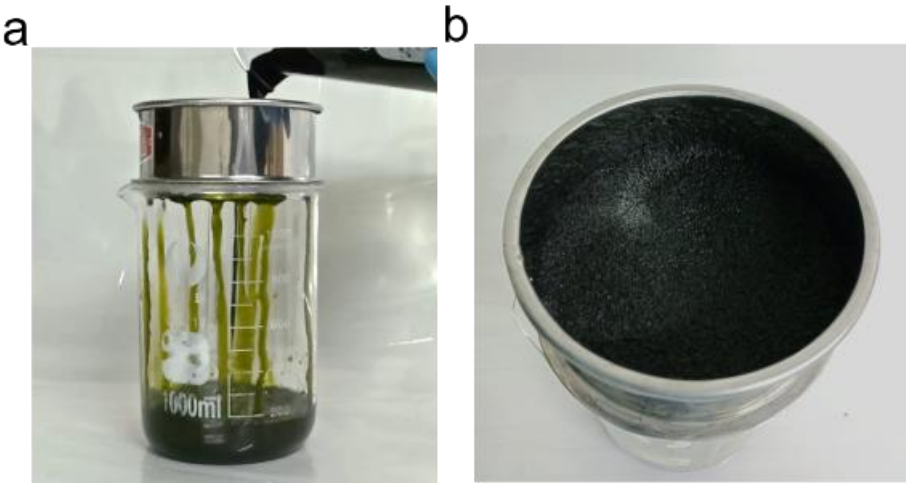

Supplementary Figure 11. (a) Mesh filtration of partially oxidized graphite to recover sulfuric acid; (b) Filter cake of the partially oxidized graphite.

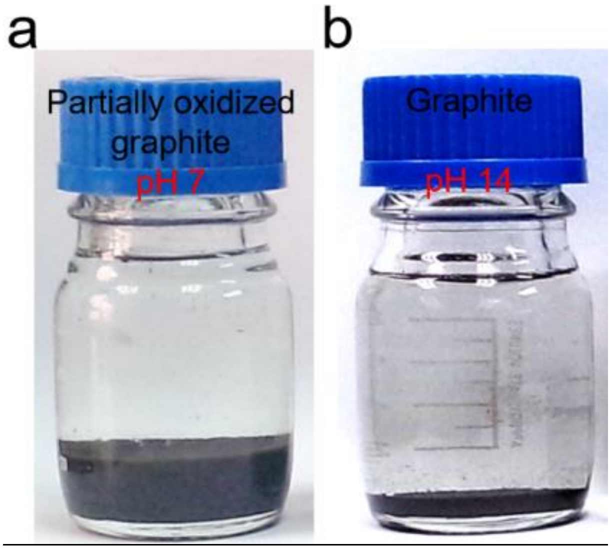

Supplementary Figure 12. Two control experiments show the precipitation of exfoliated graphene flakes. Partially oxidized graphite in neutral water (pH = 7, a) and raw graphite in alkaline solution (pH = 14, b) were exfoliated by sonication, giving graphene yields of 0.5 (a) and 0.2 wt% (b), respectively.

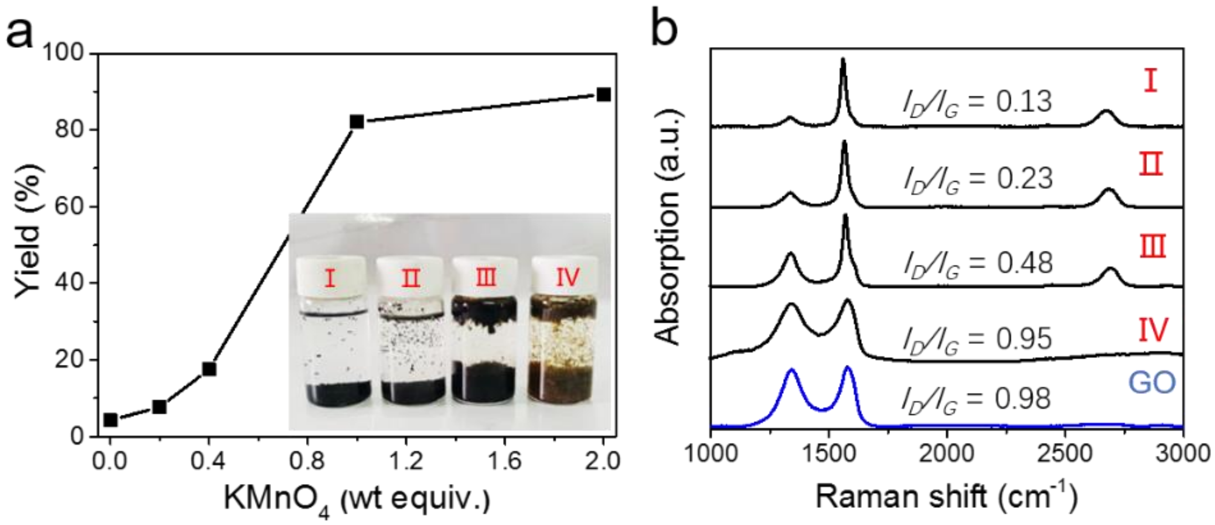

Supplementary Figure 13. (a) Graphene yields are greatly influenced by the ratio of oxidant to graphite, suggesting that appropriate oxidation is essential; (b) Raman spectra of graphite at various $KMnO_4$ ratios, together with a comparison of GO. Inset of (a), photo of graphite at different $KMnO_4$ ratio, (I) 0.4 wt. equiv., (II) 1 wt. equiv., (III) 2 wt. equiv. and (IV) 4 wt. equiv.

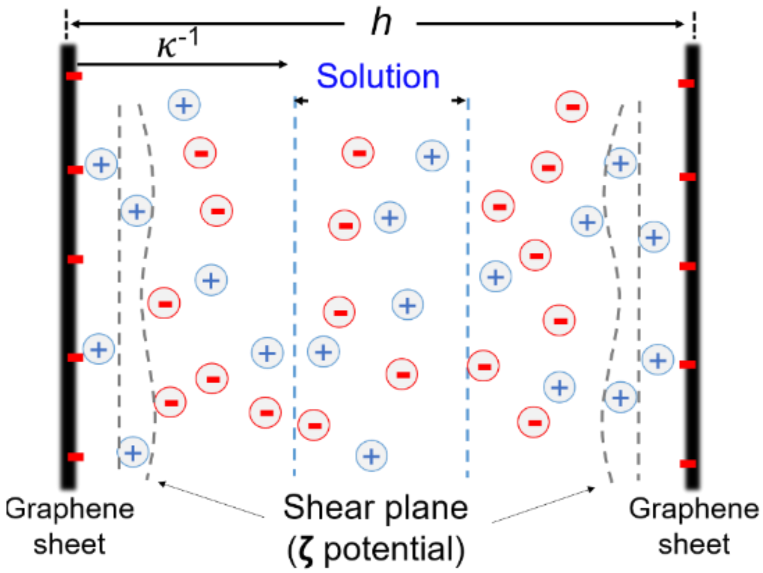

Supplementary Figure 14. Schematic illustration of the EDL model for two parallel graphene flakes in alkaline water, separated by a distance of *h*. Oxygen-containing groups on graphene are ionized, generating a negetively charged surface. The negatively charged surface and adsorbed counter ions (Na^+) form an EDL with a thickness of κ^{-1}. A slipping plane separates the mobile solution from the fluid bound on the graphene surface and is measurable experimentally (*Zeta* potential, ζ).

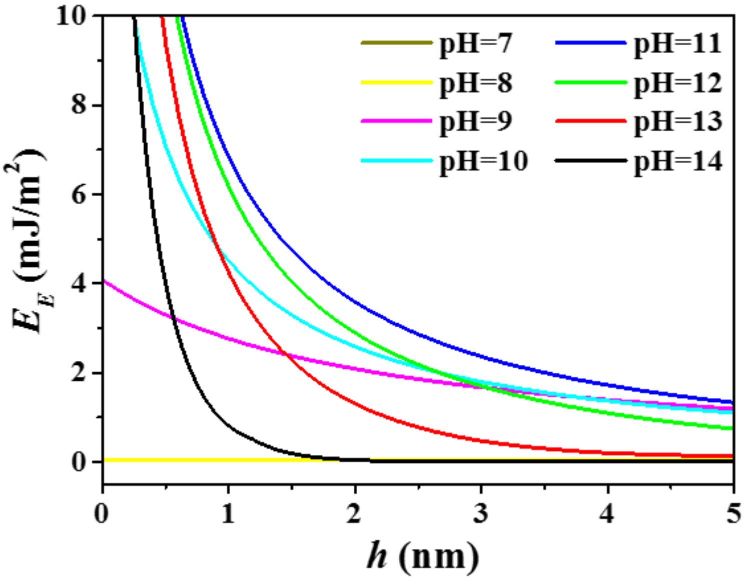

Supplementary Figure 15. E_E curves as a function of h at different pH values.

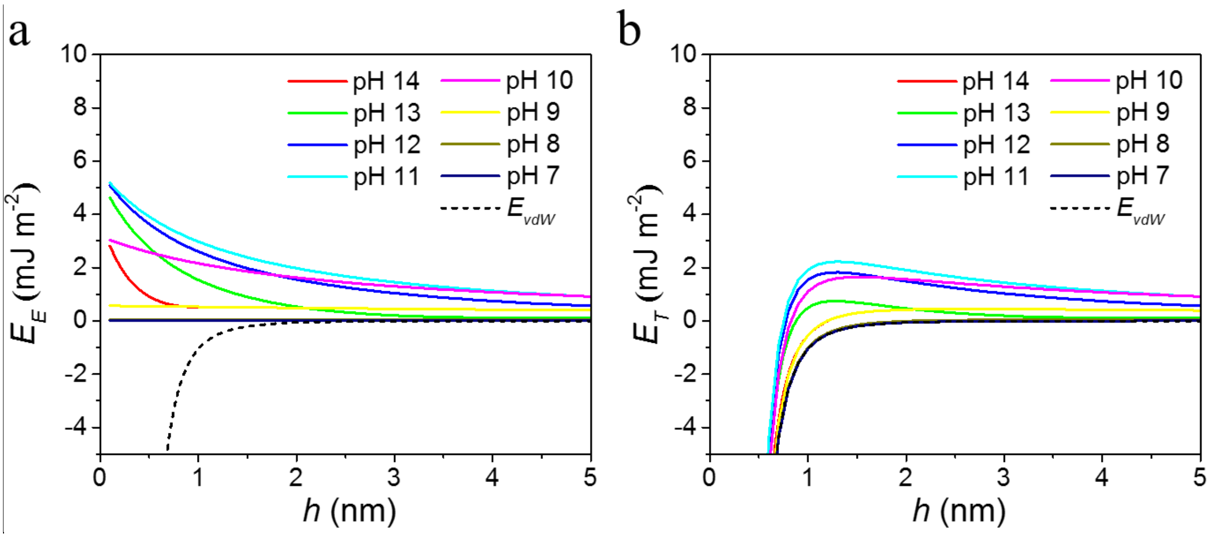

Supplementary Figure 16. E_E (a) and E_T (b) curves as a function of h for oxygen content of 1 atom%.

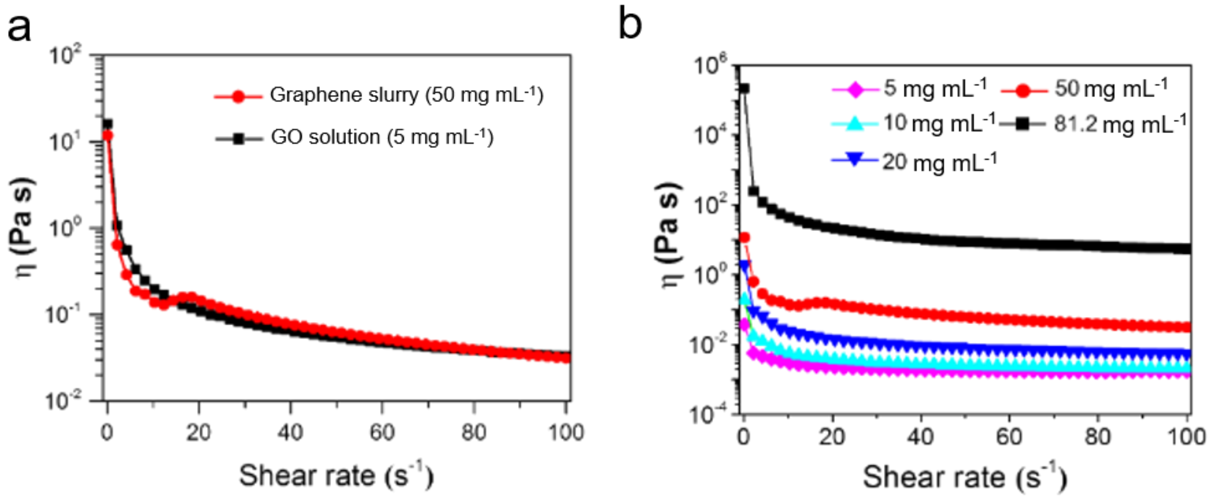

Supplementary Figure 17. (a) Shear viscosity of the resulting graphene slurry and GO solution. (b) Shear viscosity curves of different concentrations of graphene slurries, which were prepared through concentrating or diluting the graphene slurry at 50 mg mL^{-1}.

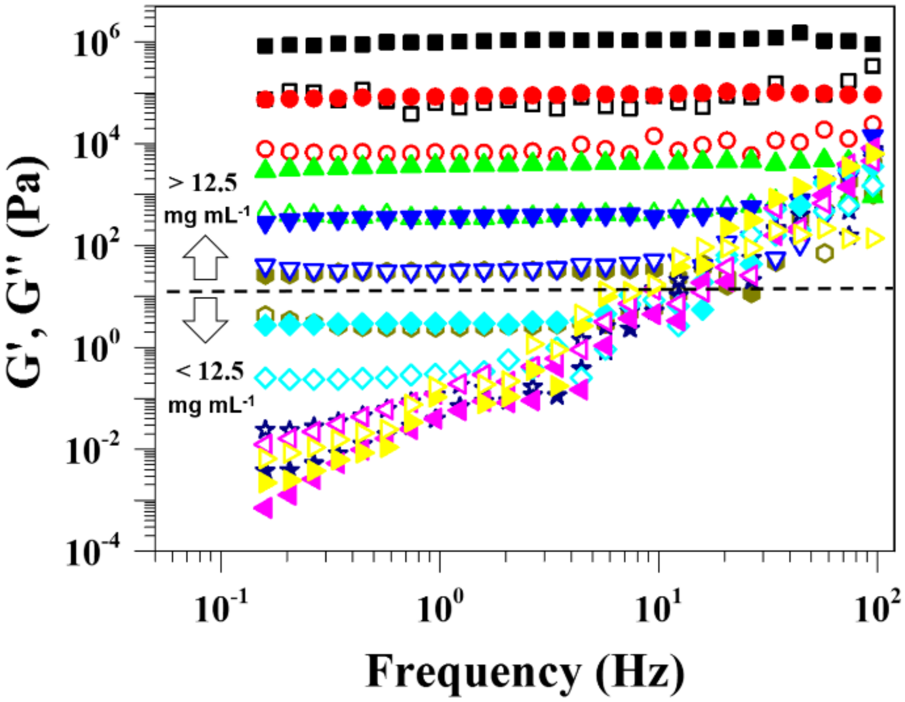

Supplementary Figure 18. Storage (G', solid dots) and loss modulus (G'', open dots) of different concentrations of graphene slurry (pH = 14): 232 mg mL^{-1} (black, 23.2 wt%), 141 mg mL^{-1} (red), 85 mg mL^{-1} (green), 50 mg mL^{-1} (blue), 25 mg mL^{-1} (dark yellow), 12.5 mg mL^{-1} (cyan), 10 mg mL^{-1} (navy), 5 mg mL^{-1} (magenta) and 1 mg mL^{-1} (yellow).

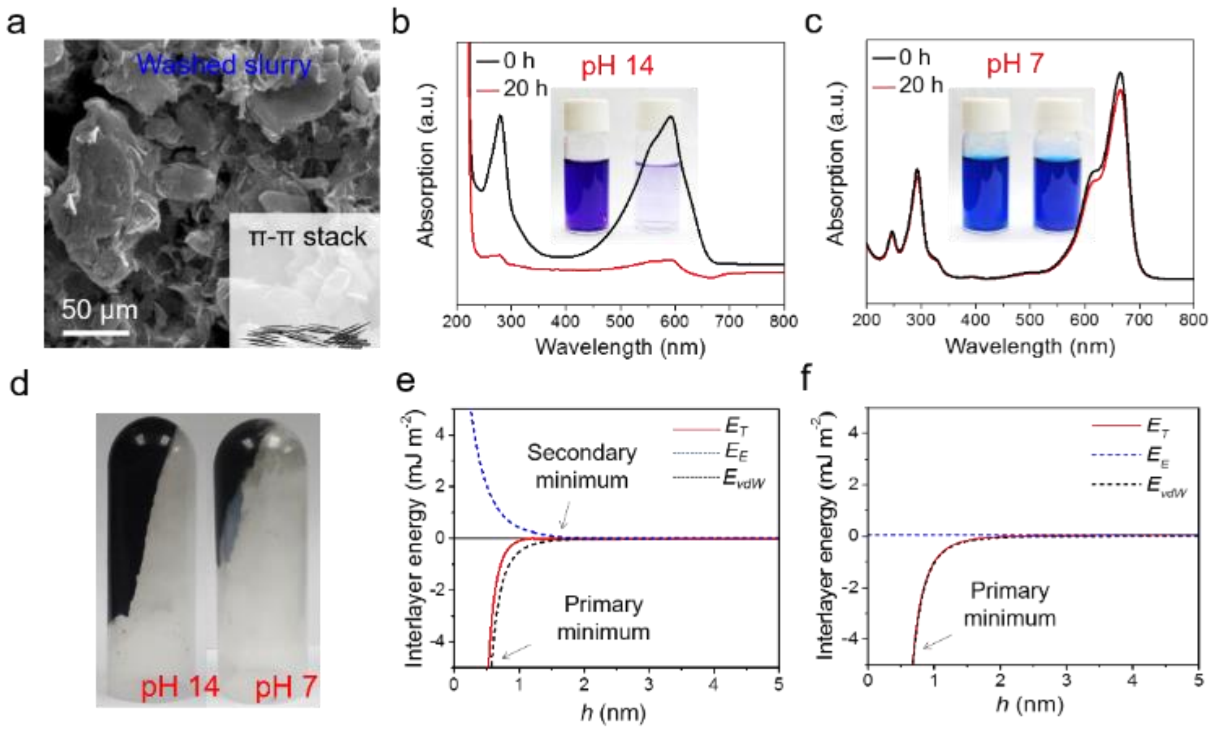

Supplementary Figure 19. (a) SEM images of the washed graphene slurry with close face-to-face structure. (b,c) MB absorption results of graphene slurries at pH = 14 and pH = 7. The adsoption quantities of MB at pH = 14 from UV-Vis spectra is 11.3 times higher than that of at pH = 7, corresponding to a specific surface area of 1240 m^2 g^{-1}. (d) Photo of graphene slurries after centrifugation at 10,000 rpm for 10 mins. Graphene slurries at pH = 14 and pH = 7 show distinct stacking densities (141 and 470 mg mL^{-1}). (e,f) The interlayer energy curves between two graphene flakes at pH = 14 (e) and pH = 7 (f).

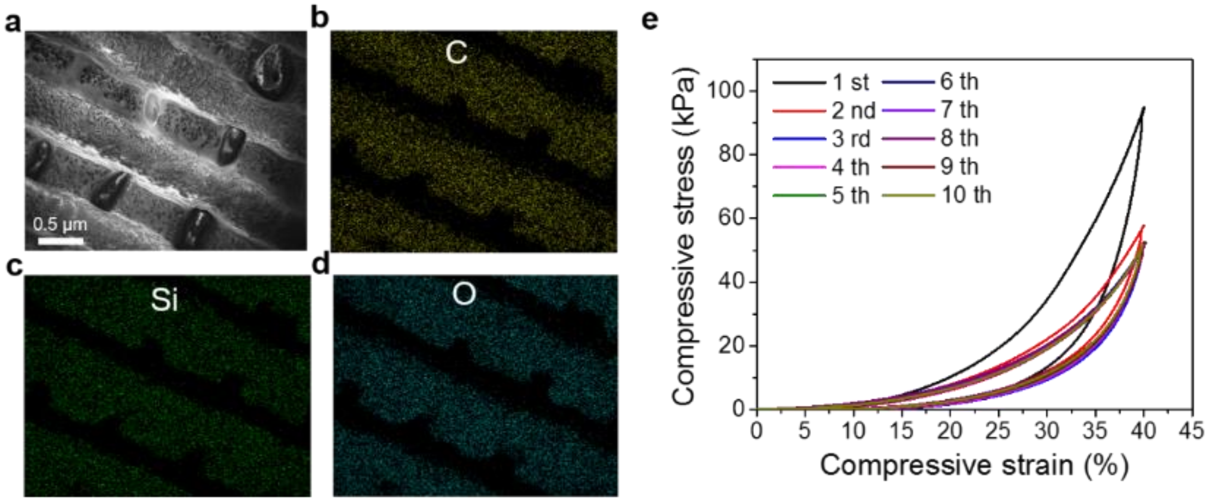

Supplementary Figure 20. (a) SEM images, corresponding elemental mapping (b-d) and (e) loading-unloading cycles of PDMS-graphene composites with 40% of structural strain.

Supplementary Table 1. Combustion-based elemental analysis of graphite and graphene

Sample	Composition / Atom% (Weight%)			
	C	N	H	O (By difference)
Graphite	93.5 (96.2)	< 0.2 (< 0.3)	4.0 (0.4)	2.3 (3.1)
Graphene	85.0 (90.5)	< 0.2 (< 0.3)	8.9 (0.8)	5.9 (8.4)

Supplementary Table 2. Comparison in graphene production and quality of our methodology with other liquid phase exfoliations

Ref.	Physical process	Solution	Graphite µm	Conc. mg mL^{-1}	Yield %	Scale g	Time h	Size µm	Thickness layer(s)	I_D/I_G	Conductivity S m^{-1}
**Ours**	**Shearing**	**H_2O at pH = 14**	**~ 500**	**50**	**82.5**	**100 (1000, 4 h)**	**2 (4 h)**	**1~6**	**1 layer, 90%**	**0.22 ~ 0.33**	**25,200 or 42,400 after reduction [a]**
1	Shearing	NMP	> 150	0.07	< 0.1	5.3	1	0.3~0.8	5~8 layers	0.18	20,000 or 40,000 after annealing
2	Microwave	Oligomeric-PF_6 salt	~ 500	25	93	0.1	0.5	1~5	1 layer, 95%	0.14	2.1 kΩ
3	Microwave	H_2SO_4 + HNO_3	~ 20	2	50	0.02	0.008	20~30	1 layer	0.45	6,600 or 19,200 after annealing
4	Electrochemistry	Inorganic salt + H_2O	--	--	85	16.3	0.5	> 5 (80%)	< 3 layers	0.25	11 Ω for graphene film
5	Electrochemistry	Reducing agents + H_2O	--	--	75	15.1	1	5~10	1~6 layers	0.1	59,000
6	Spontaneous exfoliation	H_2SO_4 + $(NH_4)_2S_2O_8$	--	12.5	~ 100	0.1	3~4	10~60	10~35 nm	~ 0.2	~ 23.4 Ω for graphene film
7	Spontaneous exfoliation	Chlorosulphonic acid	--	~ 2	--	--	48	0.3~0.9	1 layer, 70%	0.1 ~ 0.5	110,000
8	Ball-milling	Triazine derivatives	--	0.05~0.37	--	0.0075	0.5	--	Few layers	0.31 ~ 0.82	--

9	Ultrasonic	NMP	~ 500	0.01	1	~ 2	0.5	0.5~2	< 5 layers	--	6,500
10	Ultrasonic	NMP	--	1.2	4	2.31	343	< 1	< 10 layers	--	18,000 or 35,000 after annealing
11	Ultrasonic	N-octylbenzene + NMP	--	0.25	--	0.1	6	< 0.5	< 5 layers	0.3 ~ 1.9	62,500
12	Ultrasonic (intercalated by Brønsted acids)	DMF	1~100	1	~5	0.001	0.5	0.1~2	1~2 layers	< 0.1	--
13	Ultrasonic	Ionic liquid (imidazolium salt)	--	5.33	--	0.175	24	--	Few layer	~ 0.2	--
14	Ultrasonic	Pyridinium tribromide + water/ethanol	HOPG	0.04	--	0.002	0.75	0.175	1 layer, 75%	0.004	600 or 5,100 after annealing

[a.] To increase the conductivity, the dried graphene film was immersed into HI solution (57 %) at 60°C for 10 min. After repeatedly washing with water and ethanol, the resulting graphene film was vacuum-dried for electrical measurements.

Supplementary Table 3. Comparison between our approach and chemical reduction of graphene oxide

Ref	Post Reduction	Temp. °C	Time h	C/O	Thickness layer(s)	I_D/I_G	Conductivity S m^{-1}
**Ours**	**N. A.**	**--**	**--**	**16.0**	**1 layer, 90%**	**0.22 ~ 0.33**	**25,200 or 42,400 after reduction**
15	Hydrazine	100	24	10.3	--	> 1	200
16	HI	100	1	12	--	> 1	29,800
17	Na + NH_3	-78	0.5	16.6	--	> 1	350 Ω, 80% transmittance
18	Hydrazine	r. t.	~ 168	--	--	> 1	--
19	HI + acetic acid	40	40	15.3	--	> 1	30,400
20	Na + ethanol	220	72	6.4	1 layer	1.16	0.05
21	$NaBH_4$	80	1	4.8	--	1.9	82
22	Fe + HCl	r. t.	6	7.9	2-10 layers	0.32	2,300
23	Propylene carbonate	150	12	8.3	--	--	2,100
24	H_2O	180	6	5.6	1 layer	0.9	--
25	Hydrazine + DMF/H_2O	80	1	11	1 layer	> 1	1,700 or 16,000 (dried at 150 °C)
26	H_2O	95	48	6	1 layer, 65%	--	--
27	Argon	500	1	--	--	--	35,100

Supplementary Table 4. Calculated results of c, W, σ, κ, κ^{-1} and ψ_0

pH	c mol m^{-3}	$\boldsymbol{W}$	$\boldsymbol{\sigma}$ C m^{-2}	$\boldsymbol{\kappa}$ nm^{-1}	$\boldsymbol{\kappa^{-1}}$ nm	$\boldsymbol{\psi_0}$ mV
7	0.0001	0.0010	0.00037	0.00105	954.2	0.1515
8	0.001	0.0101	0.0036	0.00332	301.7	0.2083
9	0.01	0.0926	0.033	0.01048	95.42	0.2621
10	0.1	0.5050	0.181	0.03315	30.17	0.2899
11	1	0.911	0.327	0.1048	9.542	261.64
12	10	0.990	0.355	0.3315	3.017	207.65
13	100	1.000	0.359	1.038	0.9542	150.68
14	1000	1.000	0.359	3.315	0.3017	93.18

Supplementary Note 1

Calculation of electrostatic repulsion energy. A concise and precise expression for the EDL interaction is desirable in colloid and surface sciences. The Poisson-Boltzmann equation (PB) provides a theoretical foundation for calculating the EDL interaction. Here, for two parallel plate-like graphene flakes with a constant surface potential (ψ_0), ψ_0 is assumed to be unchanged, independent of the separation distance of two graphene flakes (h). The PB equation for the potential ($\psi(x)$) in distance x from graphene flake is expressed as[28,29]

$$\frac{d^2\psi}{dx^2} = \frac{2veLc}{\varepsilon_0\varepsilon_r}\sinh\frac{ve\psi}{kT} \ , \tag{1}$$

where v is the valence of electrolyte (1 for Na^+), e is the elementary electric charge (1.602×10^{-19} C), L is the Avogadro constant (6.02×10^{23}), c is the electrolyte concentration, ε_0 is the permittivity of vacuum (8.85×10^{-12} F/ m), ε_r is the relative permittivity of the solution (78.36 for water), k is the Boltzmann constant (1.38×10^{-23} J K^{-1}), and T is the absolute temperature (298 K).

Many studies have reported how to constitute the relation between x and the electrostatic repulsion energy E_E. In particular, exploiting the Debye-Hückel linear approximation to the PB equation, Hogg, Healy and Fuerstenau proposed a simplified formula (called HHF formula) to estimate the interaction between two spherical particles at constant surface potential[31], which has been widely used in colloid science. Despite good approximation to the experimental results, the HHF equation is too tedious for practical use and furthermore, accurate only at low and moderate potentials. Afterward, Ohshima proposed a novel linearization method to solve the PB equation for the interaction energy between two identical parallel plates[32]. However, this linearizing expression is accurate only near the plate surface and within a relatively low potential range. Wang reported a simplified method by using the improved Derjaguin method at high potentials ($\psi_0 \gg 1$)[30,31]. The expression of E_E is relatively accurate by dividing into two equations in strong interaction region and weak interaction region[31]. The relative error is found to be largest (~10 %) at the juncture of strong and weak interaction (where κh is 4). The two equations are written as

$$E_E = \frac{2LckT}{\kappa}\left\{2\pi^2\left(\frac{1}{\kappa h+b}\right) - \left(\frac{1}{6.9+b}\right) - \frac{1}{24\pi^2}[(\kappa h + b)^3 - 2\pi + b^3] + (\kappa h - 2\pi)\right\}, \tag{2}$$

where b is related to ψ_0 and defined by $b = 4\mathrm{e}^{\frac{-ze\psi_0}{2kT}}$, and κ is the Debye-Hückel parameter (κ^{-1} is regarded as the thickness of electrical double layer) and defined by $\kappa = (\frac{2Lce^2v^2}{\varepsilon_0\varepsilon_r kT})^{0.5}$. The relative errors of E_E is less than 10% when $\kappa h< 4.0$. Beyond the range, the error rapidly increases and another equation is derived as follows,

$$E_E = \frac{64LckT}{\kappa}(\gamma_0{}^2 \cdot e^{-kh} + \frac{1}{3}\cdot \gamma_0{}^4 \cdot e^{-2kh}) \ , \tag{3}$$

where γ_0 is defined by $\gamma_0 = \tanh(\frac{-e\psi_0}{4kT})$. Here, the error of E_E in Supplementary Equation (3) reaches the maximum (~10 %) at $\kappa h= 4.0$.

We next determine the relationship between ψ_0 and surface charge density of graphene flakes (σ). According to the boundary condition at the flake surface x= 0, ψ is expressed as

$$\left(\frac{d\psi}{dx}\right)_{x=0} = -\frac{\sigma}{\varepsilon_0 \varepsilon_r} \ . \tag{4}$$

By combining Supplementary Equations (2) and (4), we obtain the follow relation between ψ_0 and σ.

$$\sigma = \frac{2\varepsilon_0 \varepsilon_r \kappa k T}{ve} \sinh(\frac{ve\psi_0}{2kT}) \ . \tag{5}$$

Note that the value of σ can be obtained from the content and ionization degree of the oxygen functional groups (hydroxyl groups in our case) by

$$\sigma = W \times \frac{A}{S_c} \times e \ , \tag{6}$$

where W is the ionization degree of oxygen functional groups, A is the mole ratio of oxygen groups to carbon atoms in graphene flakes and S_c is defined by the divided area of one carbon atom in graphene flakes, which is half of one benzene ring's area (2.619×10^{-20} m^2). According to the results of XPS spectra, most of the oxygen atoms are from the –OH groups on graphene nanosheets and the content is ~ 5.9 atom%. To simplify the calculations, all the hydroxyl groups are regarded as phenolic hydroxyl groups in terms of its ionization behavior[32]. The results of c, W, σ, κ, $\kappa^{-1}$and ψ_0 at different pH values are presented in Supplementary Table 4.

Since the value of ψ_0 is much larger than 1 in the range of pH = 11 ~ 14, Supplementary Equations (3) and (4) are used in our study. Combining with Supplementary Equations (7) and (8), E_E curves as function of h are obtained at different pH values and presented in Supplementary Fig. 15.

Supplementary Note 2

Calculation of van der Waals attraction energy. Graphene flake can be imagined as a two-dimensional circular sheet of radius (R). According to the report by Coleman *et. al.*[33], E_{vdW} can be expressed as

$$E_{vdW} = \frac{\pi\rho^2 C}{2h^4} \ , \tag{7}$$

where ρ is the areal number density of atoms in the flake, C is the vdW energy coefficient. Since the value of $\rho^2 C$ can be given by a known condition, that is, the surface energy of graphite (γ, 70 mJ m^{-2}) by $\gamma = \frac{\pi\rho^2 C}{2\times(0.35\ \mathrm{nm})^4} = 70\ \mathrm{mJ\ m^{-2}}$, Supplementary Equation (7) is re-written as

$$E_{vdW} = \frac{\pi\rho^2 C}{2h^4} = \frac{1.05\times10^{-36}}{h^4}\ \mathrm{mJ\ m^{-2}} \tag{8}$$

Supplementary References

1. Paton, K. R. *et al.* Scalable production of large quantities of defect-free, few-layer graphene by shear exfoliation in liquids. *Nat. Mater.* **13**, 624-630 (2014);

2. Matsumoto, M., Saito, Y., Park, C., Fukushima, T. & Aida, T. Ultrahigh-throughput exfoliation of graphite into pristine ‘single-layer’ graphene using microwaves and molecularly engineered ionic liquids. *Nat. Chem.*, **7**, 730-736 (2015);

3. Chiu, P. L. *et al.* Microwave- and nitronium ion-enabled rapid and direct production of highly conductive low-oxygen graphene. *J. Am. Chem. Soc.* **134**, 5850-5856 (2012);

4. Parvez, K. *et al*. Exfoliation of graphite into graphene in aqueous solutions of inorganic salts. *J. Am. Chem. Soc*. **136**, 6083-6091 (2014);

5. Yang, S. *et al.* Organic radical-assisted electrochemical exfoliation for the scalable production of high-quality graphene. *J. Am. Chem. Soc.* **137**, 13927-13932 (2015);

6. Dimiev, A. M., Ceriotti, G., Metzger, A., Kim, N. D. & Tour, J. M. Chemical Mass Production of Graphene Nanoplatelets in ∼100% Yield. *ACS Nano* **10**, 274-279 (2016);

7. Behabtu, N. *et al.* Spontaneous high-concentration dispersions and liquid crystals of graphene. *Nat. Nanotechnol.* **5**, 406-411 (2010);

8. León, V., Rodriguez, A. M., Prieto, P., Prato, M. & Vázquez E. Exfoliation of graphite with triazine derivatives under ball-milling conditions: preparation of few-layer graphene via selective noncovalent interactions. *ACS Nano* **8**, 563-571 (2014);

9. Hernandez, Y. *et al.* High-yield production of graphene by liquid-phase exfoliation of graphite. *Nat. Nanotechnol.* **3**, 563-568 (2008);

10. Khan, U., O’Neill, A., Lotya, M., De, S. & Coleman, J. N. High-concentration solvent exfoliation of graphene. *Small* **6**, 864-871 (2010);

11. Haar1, S. *et al.* Enhancing the liquid-phase exfoliation of graphene in organic solvents upon addition of *n*-octylbenzene. *Sci. Rep.* **5**, 16684 (2015);

12. Kovtyukhova, N. I. *et al*. Non-oxidative intercalation and exfoliation of graphite by Brønsted acids. *Nat. Chem.* 6, 957-963 (2014);

13. Nuvoli, D. *et al.* High concentration few-layer graphene sheets obtained by liquid phase exfoliation of graphite in ionic liquid. *J. Mater. Chem.* **21**, 3428-3431 (2011);

14. Chen, I. P., Huang, C., Jhou. S. S. & Zhang Y. Exfoliation and performance properties of non-oxidized graphene in water. *Sci. Rep*. **4**, 3928 (2014);

15. Stankovich, S. *et. al.* Synthesis of graphene-based nanosheets via chemical reduction of exfoliated graphite oxide. *Carbon* **45**, 1558-1565 (2007);

16. Pei, S. F., Zhao, J. P., Du, J. H., Ren, W. C. & Cheng, H. M. Direct reduction of graphene oxide films into highly conductive and flexible graphene films by hydrohalic acids. *Carbon* **48**, 4466-4474 (2010);

17. Feng, H., Cheng, R., Zhao, X., Duan, X. & Li, J. A low-temperature method to produce highly reduced graphene oxide. *Nat. Commun.* **4**, 1539 (2013);

18. Tung, V. C., Allen, M. J., Yang, Y. & Kaner, R. B. High-throughput solution processing of large-scale graphene. *Nat. Nanotechnol.* **4**, 25-29 (2009);

19. Moon, I. K., Lee, J., Ruoff, R. S. & Lee, H. Reduced graphene oxide by chemical graphitization. *Nat. Commun.* **1**, 73 (2010);

20. Choucair, M., Thordarson, P. & Stride, J. A. Gram-scale production of graphene based on solvothermal synthesis and sonication. *Nat. Nanotechnol*. **4**, 30-33 (2009);

21. Gao, W., Alemany, L. B., Ci, L. J. & Ajayan, P. M. New insights into the structure and reduction of graphite oxide. *Nat. Chem.* **1**, 403-408 (2009);

22. Fan, Z. J. *et al.* Facile synthesis of graphene nanosheets via Fe reduction of exfoliated graphite oxide. *ACS Nano* **5**, 191-198 (2011);

23. Zhu, Y. *et al*. Exfoliation of graphite oxide in propylene carbonate and thermal reduction of the resulting graphene oxide platelets. *ACS Nano* **4**, 1227-1233 (2010);

24. Zhou, Y., Bao, Q. L., Tang, L. A. L., Zhong, Y. L. & Loh, K. P. Hydrothermal dehydration for the "green" reduction of exfoliated graphene oxide to graphene and demonstration of tunable optical limiting properties. *Chem. Mater.* **21**, 2950-2956 (2009);

25. Park, S. *et al.* Colloidal suspensions of highly reduced graphene oxide in a wide variety of organic solvents. *Nano Lett.* **9**, 1593-1597 (2009);

26. Liao, K. *et al*. Aqueous only route toward graphene from graphite oxide. *ACS Nano* **5**, 1253-1258 (2011);

27. Chen, H. Q., Muller, M. B., Gilmore, K. J., Wallace, G. G. & Li, D. Mechanically strong, electrically conductive, and biocompatible graphene paper. *Adv. Mater.* **20**, 3557-3561 (2008);

28. Hogg, R., Healy, T. W. & Fuerstenau, D. W. Mutual coagulation of colloidal dispersions. *Trans. Faraday Soc.*, **62**, 1638-1651 (1966);

29. Ohshima, H. Approximate expression for the potential energy of double-layer interaction between two parallel similar plates with constant surface potential. *Colloid Surface A* **146**, 213-216 (1999);

30. Luo, G. X., Feng, R. J., Jin, J. & Wang, H. P. Strong interaction of colloidal particles: an extension of Langmuir's method. *Langmuir* **17**, 2167-2171 (2001);

31. Luo, G. X., Feng, R. J.; Jin, J. & Wang, H. P. Strong and weak interactions of colloidal particles with high surface potentials. *J. Colloid Interf. Sci.* **241**, 81-88 (2001);

32. Konkena, B. & Vasudevan, S. Understanding aqueous dispersibility of graphene oxide and reduced graphene oxide through pK_a measurements. *J. Phys. Chem. Lett*. **3**, 867-872 (2012);

33. Lotya, M. *et al.* Liquid phase production of graphene by exfoliation of graphite in surfactant/water solutions. *J. Am. Chem. Soc.* **131**, 3611-3620 (2009).
