# Supplementary material for: A non-dispersion strategy for large-scale production of ultra-high concentration graphene slurries in water
Source: Nat Commun. 2018 Jan 8;9:76. doi: 10.1038/s41467-017-02580-3 (PMC5758749; doi:10.1038/s41467-017-02580-3)
Supplement: Supplementary file 3 — Description of Additional Supplementary Files [file 41467_2017_2580_MOESM3_ESM.pdf]

Description of Additional Supplementary Files

File Name: Supplementary Movie 1
Description: The Recovery of Concentrated Sulfuric Acid by Filtration.
